# GIVE A PIECE OF YOUR MIND: A CONTENT ANALYSIS OF A FACEBOOK SUPPORT GROUP FOR DEMENTIA CAREGIVERS

**DOI:** 10.1093/geroni/igz038.3128

**Published:** 2019-11-08

**Authors:** Jenny A Lagervall, Madeline R Lag, Sophie Brickman, Rebecca E Ingram, Leilani Feliciano

**Affiliations:** University of Colorado Colorado Springs, Colorado Springs, Colorado, United States

## Abstract

The online environment offers individuals a means of obtaining information, support, and social connection. Older adults are growing users of the internet1. Online support groups (e.g., Facebook groups) have been found to provide health-related information and encourage mental well-being2. They may be particularly advantageous for caregivers of individuals diagnosed with dementia, as it is difficult to leave loved ones with dementia alone. However, the mechanisms by which online support groups engage caregivers, and the content of support, have yet to be explored. In the current study, content from 100 posts from a private Facebook caregiver support group were evaluated for gender of post author, relationship to the person receiving care, distress, emotional tone, grief reaction, caregiver burden, and coping strategy. Results indicated that caregiver distress was associated with the presence of grief reactions, negative emotional tone, and higher caregiver burden. Utilizing venting as a coping strategy was associated with higher caregiver burden, similar to what is observed in a traditional in-person support group. Online communication for caregivers may provide an indication of caregivers’ psychological well-being, as specific coping strategies and grief reactions indicated higher levels of caregiver burden and distress. Research on interventions for dementia caregivers may benefit from a focus on online social support as a means of accessing caregivers and treatment delivery.

